# Early changes in CT-derived quantitative flow ratio after transcatheter aortic valve replacement: a serial coronary physiological assessment

**DOI:** 10.3389/fcvm.2026.1902728

**Published:** 2026-10-09

**Authors:** Lieyou Li, Yonghui Yu, Xinjie Zeng, Lijun Luo, Zhongwei Shi, Linyan Chen, Feng Hu, Lin Fan

**Affiliations:** Department of Cardiology, Fujian Medical University Union Hospital, Fujian Cardiovascular Medicine Center, Fujian Institute of Coronary Artery Disease, Fujian Cardiovascular Research Center, Fujian Medical University Heart Center, Fujian, China

**Keywords:** aortic valve stenosis, computed tomography angiography, coronary artery disease, quantitative flow ratio, transcatheter aortic valve replacement

## Abstract

**Aims:**

Transcatheter aortic valve replacement (TAVR) is an established treatment for severe aortic valve stenosis, but its effects on coronary physiology remain incompletely understood. CT-derived quantitative flow ratio (CT-QFR) enables non-invasive assessment of coronary physiology. This study aimed to investigate the early changes in CT-QFR following TAVR.

**Methods:**

This retrospective study included consecutive patients with severe aortic stenosis who underwent coronary computed tomography angiography before and within 7 days after TAVR. CT-QFR was calculated for all analyzable coronary vessels. Longitudinal changes in CT-QFR were evaluated using linear mixed-effects models with patient identifier as a random effect.

**Results:**

A total of 109 patients (324 coronary vessels) were analyzed. Plaque volume, plaque burden, and minimal lumen diameter remained unchanged after TAVR, whereas transvalvular pressure gradients decreased significantly. Overall, CT-QFR increased modestly following TAVR (*β*=0.018, *P* < 0.001). Significant increases were observed in the left anterior descending and right coronary arteries, whereas no significant change was detected in the left circumflex artery. Vessels with baseline %DS ≥50% demonstrated greater CT-QFR increases than those with less severe stenosis (interaction *β*=0.104, *P* < 0.001). Likewise, vessels with baseline CT-QFR <0.80 showed significantly larger post-TAVR increases than vessels with baseline CT-QFR ≥0.80 (interaction *β*=0.117, *P* < 0.001).

**Conclusion:**

TAVR was associated with modest early increases in CT-QFR, particularly in vessels with greater anatomical stenosis or baseline physiological impairment. These findings suggest that coronary physiological assessment in severe aortic stenosis may be influenced by valve intervention and should be interpreted cautiously during the peri-TAVR period.

## Graphical Abstract

**Figure:**
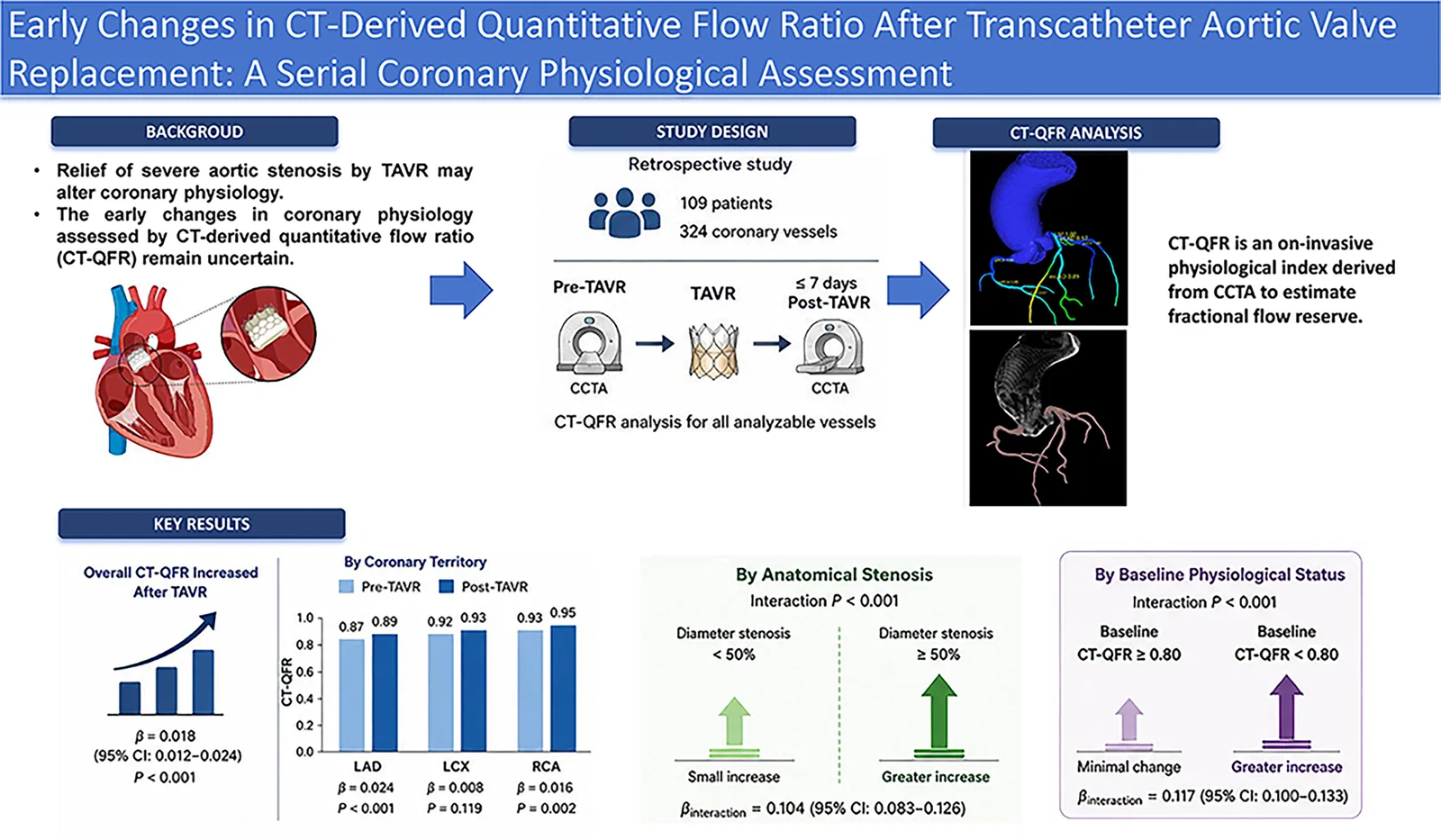

## 1 Introduction

Severe aortic valve stenosis (AVS) is a common and high-risk valvular heart disease, traditionally managed by surgical aortic valve replacement (1, 2). In recent years, transcatheter aortic valve replacement (TAVR) has emerged as a minimally invasive alternative, initially for high-surgical-risk patients and now expanding to intermediate- and low-risk populations (3, 4). However, the effects of TAVR on coronary physiology remain incompletely understood. Although coronary obstruction is a recognized procedural complication, more subtle changes in coronary hemodynamics following relief of valvular stenosis have received considerably less attention (5).

Coronary physiological assessment is increasingly used to guide the management of concomitant coronary artery disease in patients undergoing TAVR. Although invasive fractional flow reserve (FFR) remains the reference standard for physiological lesion assessment, routine invasive physiological assessment is not always feasible in the peri-TAVR setting. CT-derived quantitative flow ratio (CT-QFR) has emerged as a validated non-invasive alternative, showing good agreement with invasive FFR (6–10). Given that coronary computed tomography angiography (CCTA) is routinely performed before TAVR and increasingly incorporated into post-procedural follow-up, CT-QFR provides a unique opportunity to evaluate serial changes in coronary physiology without additional testing.

Several invasive studies have suggested that coronary physiological indices may change after relief of severe aortic stenosis (11, 12). However, evidence regarding serial CT-QFR assessment before and shortly after TAVR remains limited. Therefore, this study aimed to evaluate early changes in CT-QFR following TAVR using serial coronary CT angiography and to investigate whether these changes vary according to coronary territory, anatomical lesion severity, and baseline physiological status.

## 2 Methods

### 2.1 Patient selection

Consecutive patients with severe AVS who underwent successful TAVR between August 2019 and August 2024 were enrolled. All participants underwent preprocedural CCTA and at least one postprocedural CCTA examination within 1 week after TAVR. The patient enrollment flowchart is presented in Figure S1. This study was approved by the Institutional Ethics Committee of Fujian Medical University Union Hospital (Approval No.2025KY637) in accordance with the Declaration of Helsinki. Written informed consent was obtained from all participants.

#### 2.1.1 Inclusion criteria

According to guideline criteria, severe AVS was defined on transthoracic echocardiogram by a reduced valve area (< 1.0 cm^2^ or indexed < 0.6 cm^2^/m^2^) alongside elevated flow indices (mean gradient ˃ 40mmHg, peak velocity > 4.0 m/s, velocity ratio < 0.25) (13). All patients underwent assessment of hemodynamic and functional parameters (aortic gradients, valvular areas, and left ventricular function) before and after valve implantation. The indication for transcatheter aortic valve replacement was determined following a discussion by our institution's multidisciplinary Heart Team.

#### 2.1.2 Exclusion criteria

Patients were excluded if they met any of the following criteria: absence of informed consent; history of prior myocardial infarction; previous coronary intervention or tandem lesions within the same coronary artery; predominant aortic regurgitation as the primary valvular lesion; presence of unstable angina, acute myocardial infarction, or severe heart failure; or residual aortic regurgitation of ≥ grade 2 following TAVR.

### 2.2 TAVR procedures

Transcatheter aortic valve replacement (TAVR) was performed in a dedicated hybrid catheterization laboratory under fluoroscopic guidance using a digital subtraction angiography system (Artis Zee Ceiling, Siemens Healthineers, Forchheim, Germany, or NeuAngio 30C, Neusoft Medical Systems Co., Ltd., Shenyang, China). Device selection, procedural planning, and implantation techniques were based on preprocedural imaging findings and were performed according to current clinical guidelines and multidisciplinary heart team consensus. All procedures were performed by experienced interventional cardiologists following standardized institutional protocols.

### 2.3 CTA acquisition

Imaging was performed on a SOMATOM Force scanner (Siemens Healthineers, Germany), a third-generation dual-source CT system. Assessment of the overall coronary calcification burden was first conducted via the Agatston scoring method. For the coronary CTA itself, a prospective ECG-triggering technique was employed, with image acquisition covering a window from 35% to 75% of the R-R interval. Automated tube voltage and current modulation was applied to reduce radiation exposure. More details regarding acquisition parameters are given in the Supplementary file 1.

### 2.4 CT-QFR acquisition

The CT-QFR analysis was conducted using a dedicated research software platform (Cta-Plus, v2.0; Pulse Medical Imaging Technology, China), which is founded on quantitative flow ratio technology. This algorithm's diagnostic accuracy has been previously validated against invasive fractional flow reserve (FFR) as the gold standard (8, 9). Computational methods and the process for onsite analysis are detailed in the online supplemental materials. Vessel-specific CT-QFR values were measured from the ostium to the distal segment of left anterior descending artery (LAD), left circumflex artery (LCX), and right coronary artery (RCA). For both pre-procedural and post-procedural CT-QFR analyses of the same vessel, measurements were performed with consistent starting and ending points. Representative images of coronary CT-QFR analysis were shown in Figure 1.

**Figure 1:**
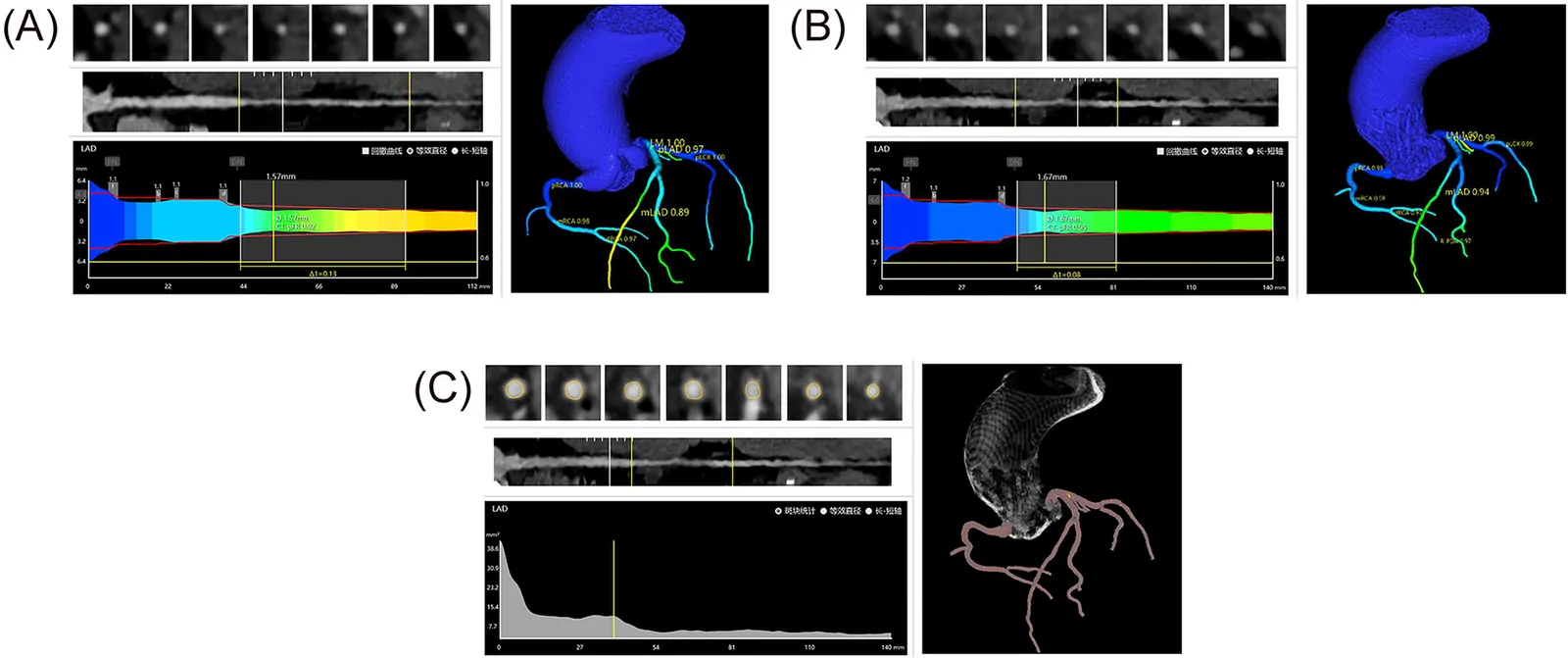
Representative images of coronary CT-QFR analysis in a 66-year-old female patient with severe aortic stenosis and mild coronary stenosis; **(A)** CT-QFR analysis of LAD before TAVR; **(B)** CT-QFR analysis of LAD after TAVR; **(C)** plaque characterization analysis of LAD.

### 2.5 CCTA-based plaque analysis

All acquired CCTA datasets were exported to an offline post-processing workstation (syngo.via, VB20A; Siemens Healthineers) for analysis. The highest-quality images were chosen for the manual evaluation of diameter stenosis. The percent diameter stenosis (%DS) was computed according to the standard formula: (reference diameter-minimal lumen diameter)/reference diameter   ×   100%.

Subsequent quantitative plaque assessment was carried out with a specialized research application (Coronary Plaque Analysis, v2.0; Siemens Healthineers), which enables semi-automated plaque quantification. The determination of plaque characteristics (fibrous, calcified, lipid plaque, mixed) relied on the adaptive threshold method and the deep machine learning principle (medium membrane segmentation model) for quantitative analysis.

Two cardiovascular radiologists, each with at least 3 years of experience in cardiac imaging and blinded to the study protocol, independently analyzed the lesions. Disagreements between the observers were resolved by consensus.

### 2.6 Statistical analysis

Continuous variables are presented as mean ± standard deviation or median (interquartile range [IQR]) according to their distribution, whereas categorical variables are expressed as counts and percentages. Because multiple coronary vessels were analyzed within the same patient and repeated CT-QFR measurements were obtained before and after TAVR, vessel-level observations were not statistically independent. Therefore, linear mixed-effects models were used to account for within-patient clustering and repeated measurements over time. Patient identifier was included as a random effect, whereas time point (pre-TAVR vs. post-TAVR), coronary territory (LAD, LCX, and RCA), baseline diameter stenosis category (%DS ≥50% vs. <50%), and baseline CT-QFR category (CT-QFR <0.80 vs. ≥0.80) were treated as fixed effects. The overall effect of TAVR on CT-QFR values was assessed using a mixed-effects model with time as the primary explanatory variable. Interaction terms were subsequently introduced to evaluate whether CT-QFR changes differed according to coronary territory, baseline stenosis severity, or baseline functional status. Correlations between CT-QFR and diameter stenosis were evaluated using Pearson correlation analysis and expressed as Pearson's correlation coefficients. A two-sided *P* value <0.05 was considered statistically significant. Statistical analyses were performed using Stata/SE version 18.0 (StataCorp, College Station, TX, USA) and IBM SPSS Statistics version 26.0 (IBM Corp., Armonk, NY, USA).

## 3 Results

### 3.1 Baseline characteristics

This study included 109 patients with severe AVS, with 324 vessels analyzed. The mean age was 72.0 ± 5.9 years, and 65 patients (59.6%) were male. The predominant presenting symptoms were angina (66.1%) and dyspnea (79.8%). Regarding concomitant diseases, hypertension and diabetes mellitus were observed in 68 (62.4%) and 22 (20.2%) patients, respectively. Severe AVS with concomitant severe regurgitation was present in 17 patients (15.6%). Among the cohort, 14 patients (12.8%) had chronic kidney disease and 11 (10.1%) had atrial fibrillation. Preoperatively, 82.6% of patients were in NYHA class 3–4. The mean STS risk score was 6.2 ± 1.8, with a median proBNP level of 1,724.0 pg/mL (IQR 484.0−4,959.0). Regarding TAVR procedures, general anesthesia was used in 88 (80.7%) patients, pre-balloon dilatation was performed in 78(71.6%), and post-dilation in 38 (34.9%). Preoperative CT measurements demonstrated a mean aortic annular diameter of 25.0 ± 2.7 mm, and a median aortic valve calcium volume of 638.81 mm^3^ (IQR: 319.92−1,088.70). Among the 324 vessels analyzed, 23 (7.1%) demonstrated ≥50% diameter stenosis. Severe stenosis (≥70%) was observed in only one right coronary artery (RCA). The clinical characteristics of participants are summarized in Table 1.

**Table 1:** Baseline characteristics of participants.

Demographics	
Age, years	72.0 ± 5.9
BMI, kg/m^2^	23.37 ± 3.12
Male, n(%)	65 (59.6%)
Clinical manifestation and concomitant diseases	
Dizzy, n (%)	9 (8.3%)
Angina, n (%)	72 (66.1%)
Dyspnea, n (%)	87 (79.8%)
Syncope, n (%)	9 (8.3%)
Concomitant diseases	
History of smoking, n (%)	27 (24.8%)
History of drinking, n (%)	9 (8.3%)
Hypertension, n (%)	68 (62.4%)
Diabetes mellitus, n (%)	22 (20.2%)
Dyslipidemia, n (%)	11 (10.1%)
History of stroke, n (%)	7 (6.4%)
Chronic kidney diseases, n (%)	14 (12.8%)
Atrial fibrillation, n (%)	11 (10.1%)
Functional classification and prior surgical risk	
SBP, mmHg	126.0 ± 19.9
DBP, mmHg	69.2 ± 11.4
Heart rate, beats/min	76.8 ± 11.8
NYHA grading	
1	1 (0.9%)
2	18 (16.5%)
3	80 (73.4%)
4	10 (9.2%)
STS scoring system	6.2 ± 1.8
Baseline blood values	
NT-proBNP (pg/ml)	1,724.0 (484.0–4,959.0)
Hemoglobin (g/l)	122.8 ± 18.5
Baseline echocardiographic features	
Severe aortic stenosis, n (%)	109 (100%)
Aortic regurgitation, n (%)	
None	34 (31.2%)
Mild	27 (24.8%)
Moderate	31 (28.4%)
Severe	17 (15.6%)
Aortic CT measurements	
Aortic annulus mean diameter, mm	25.0 ± 2.7
Calcium volume, mm^3^	638.81 (319.92,1088.70)
LVOT mean diameter, mm	26.0 ± 3.9
Ascending aorta mean diameter, mm	36.6 ± 5.8
STJ mean diameter, mm	30.7 ± 5.0
LCA height, mm	15.1 ± 3.8
RCA height, mm	19.1 ± 3.2
Procedural characteristics	
Anesthesia method, n (%)	
General anesthesia	88 (80.7%)
Sedation induction and local anesthesia	21 (19.3%)
Artificial valve diameter	26.3 ± 3.4
Coronary protection, n (%)	2 (1.8%)
ECMO assisted, n (%)	1 (0.9%)
Pre-balloon dilatation, n (%)	78 (71.6%)
Post-balloon dilatation, n (%)	38 (34.9%)
Perivalvular leakage, n (%)	
None	31 (28.4%)
Mild	78 (71.6%)
Coronary vessel characteristics	
Total vessels analyzed, n	324
LAD, n (%)	108 (33.3%)
LCX, n (%)	107 (33.0%)
RCA, n (%)	109 (33.6%)
Baseline CT-QFR <0.80, n (%)	33 (10.2%)
Diameter stenosis ≥50%, n (%)	23 (7.1%)
Diameter stenosis ≥70%, n (%)	1 (0.3%)

LVOT, left ventricular outflow tract; STJ, sinotubular junction; LCA, left coronary artery; RCA, right coronary artery; SBP, systolic blood pressure; DBP, diastolic blood pressure.

Postoperatively, no significant changes were observed in LVEF (60.94 ± 14.06% vs. 61.89 ± 18.08%, *p* = 0.735), LVFS (33.54 ± 9.53% vs. 32.68 ± 10.70%, *p* = 0.273), or LVEDD (52.53 ± 8.81 mm vs. 52.52 ± 8.70 mm, *p* = 0.998). In contrast, both PPG (89.20 ± 27.99 mmHg vs. 24.95 ± 12.10 mmHg, *p* < 0.001) and MPG (56.6 ± 18.80 mmHg vs. 14.08 ± 7.19 mmHg, *p* < 0.001) decreased significantly.

### 3.2 Early CT-QFR changes following TAVR

Plaque characteristic analysis revealed no significant differences in the total plaque volume, plaque burden, or MLD between pre- and post-TAVR assessments in the LAD (volume: 193.78 ± 249.48 vs. 191.1 ± 196.03mm3, *p* = 0.875; burden: 46.9 ± 27.72 vs. 49.92 ± 25.58%, *p* = 0.125 or MLD: 2.69 ± 1.24 vs. 2.59 ± 1.26 mm, *p* = 0.593), LCX (volume: 56.35 ± 86.44 vs. 52.45 ± 74.90mm3, *p* = 0.563; burden: 36.22 ± 26.39 vs. 37.02 ± 24.64%, *p* = 0.697 or MLD: 2.22 ± 1.85 vs. 2.69 ± 1.09 mm, *p* = 0.516), and RCA(volume: 184.93 ± 282.67 vs. 156.29 ± 256.19mm3, *p* = 0.107; burden: 42.66 ± 29.57 vs. 38.37 ± 28.74%, *p* = 0.127; MLD: 2.21 ± 0.99 vs. 2.55 ± 0.74 mm, *p* = 0.137) ，as shown in Table 2. In contrast, CT-QFR values demonstrated significant early changes following TAVR. CT-QFR increased significantly in the LAD (0.89 ± 0.06 vs. 0.87 ± 0.08; *p* < 0.001) and RCA (0.95 ± 0.05 vs. 0.93 ± 0.07; *p* = 0.002). Whereas no significant change was observed in the LCX (0.93 ± 0.05 vs. 0.92 ± 0.06; *p* = 0.119), as shown in Table 3. Linear mixed-effects modeling demonstrated a significant overall effect of TAVR on CT-QFR values, with an estimated increase of 0.018 following the procedure(*β*=0.018, 95%CI: 0.012−0.024, *p* < 0.001), as shown in Table 4.

**Table 2:** Changes in coronary plaque characteristics and echocardiographic parameters before and after TAVR.

Variable	Pre-TAVR	Post-TAVR	P
CT-derived plaque characteristics			
Total plaque volume (LAD), mm^3^	191.1 ± 196.03	193.78 ± 249.48	0.875
Plaque burden (LAD), %	49.92 ± 25.58	46.9 ± 27.72	0.125
MLD (LAD), mm	2.59 ± 1.26	2.69 ± 1.24	0.593
Total plaque volume (LCX), mm^3^	52.45 ± 74.90	56.35 ± 86.44	0.563
Plaque burden (LCX), %	37.02 ± 24.64	36.22 ± 26.39	0.697
MLD (LCX), mm	2.69 ± 1.09	2.22 ± 1.85	0.516
Total plaque volume (RCA), mm^3^	156.29 ± 256.19	184.93 ± 282.67	0.107
Plaque burden (RCA), %	38.37 ± 28.74	42.66 ± 29.57	0.127
MLD (RCA), mm	2.55 ± 0.74	2.21 ± 0.99	0.137
Echocardiographic characteristics			
LVEDD (mm)	52.52 ± 8.70	52.53 ± 8.81	0.998
LVFS (%)	32.68 ± 10.70	33.54 ± 9.53	0.273
LVEF (%)	61.89 ± 18.08	60.94 ± 14.06	0.735
PPG, mmHg	89.20 ± 27.99	24.95 ± 12.10	<0.001
MPG, mmHg	56.56 ± 18.80	14.08 ± 7.19	<0.001

MLD, minimal lumen diameter; QFR, quantitative flow ratio; LVEDD, left ventricular end-diastolic dimension; PPG, peak pressure gradient; MPG, mean pressure gradient; LVFS, Left ventricular fractional shortening.

**Table 3:** Linear mixed-effects model analysis of pre- and post-TAVR CT-QFR changes.

Coronary artery	Pre-TAVR	Post-TAVR	*β*	95% CI	P
CT-QFR of LAD	0.87 ± 0.08	0.89 ± 0.06	0.024	(0.014, 0.034)	<0.001
CT-QFR of LCX	0.92 ± 0.06	0.93 ± 0.05	0.008	(−0.002, 0.018)	0.119
CT-QFR of RCA	0.93 ± 0.07	0.95 ± 0.05	0.016	(0.006, 0.026)	0.002

**Table 4:** Linear mixed-effects models evaluating longitudinal changes in CT-QFR following TAVR.

Model/Variable	β	95%CI	p
Panel A. Overall longitudinal change			
Post-TAVR (vs. pre-TAVR)	0.018	0.012, 0.024	<0.001
Panel B. Interaction with baseline physiological status			
Post-TAVR	0.001	0.001, 0.012	0.011
Baseline CT-QFR <0.80	−0.203	−0.222, −0.183	<0.001
Post-TAVR × Baseline CT-QFR <0.80	0.117	0.100, 0.133	<0.001
Panel C. Interaction with coronary territory			
Post-TAVR	0.014	0.006, 0.021	<0.001
LAD (reference: non-LAD)	−0.059	−0.070, −0.047	<0.001
Post-TAVR × LAD	0.012	−0.004, 0.025	0.058
Panel D. Interaction with anatomical stenosis			
Post-TAVR	0.011	0.005, 0.016	<0.001
Diameter stenosis ≥50%	−0.133	−0.161, −0.104	<0.001
Post-TAVR × Diameter stenosis ≥50%	0.104	0.083, 0.126	<0.001

Data are presented as β coefficients with 95% confidence intervals (CI). Linear mixed-effects models included patient identifier as a random intercept to account for repeated vessel-level measurements. Fixed effects included time (pre- vs. post-TAVR) and the corresponding interaction terms. A two-sided *P* < 0.05 was considered statistically significant. LCA: left coronary artery; RCA: right coronary artery; LCX:left circumflex artery.

### 3.3 Influence of baseline stenosis severity

Baseline %DS was strongly associated with CT-QFR values (r = −0.74, *P* < 0.001) and showed good accuracy for predicting positive CT-QFR (AUC=0.86; 95% CI: 0.80–0.93) (Figure S2). Among 23 coronary vessels with %DS ≥50%, 11 had positive CT-QFR (<0.8) before TAVR, and 6 of these (54.5%) converted to negative CT-QFR ≥ 0.8 post-TAVR. Mixed-effects analysis demonstrated that vessels with diameter stenosis ≥50% exhibited significantly lower CT-QFR values than vessels with less severe stenosis (*β*=−0.133, 95% CI:−0.161 to −0.104, *P* < 0.001), as shown in Table 4. Importantly, a significant interaction between TAVR and stenosis severity was observed (*β*=0.104, *p* for Interaction <0.001), indicating that vessels with greater anatomical stenosis experienced larger increases in CT-QFR following TAVR (Figure 2).

**Figure 2:**
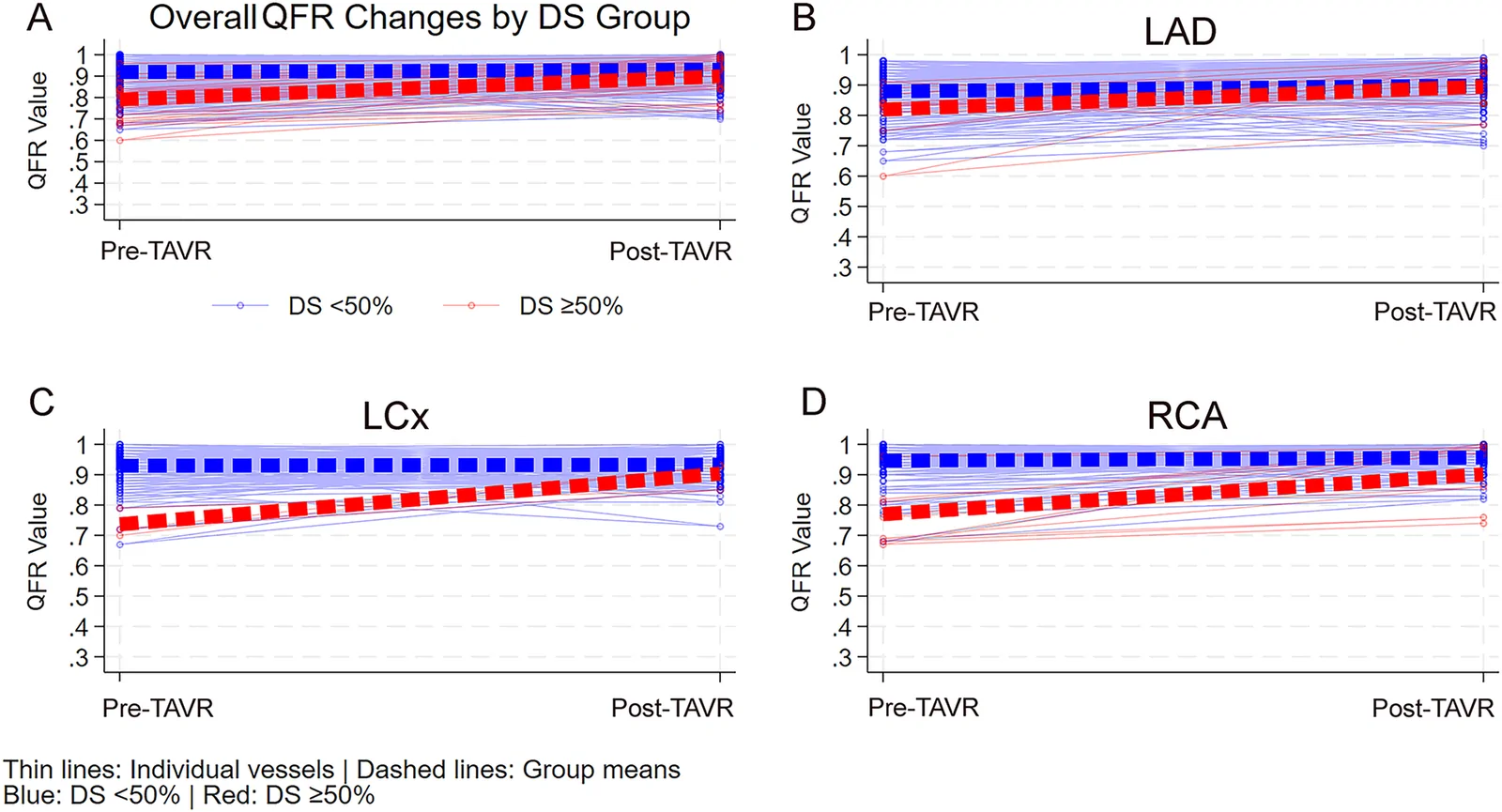
**(A)** overall changes in CT-QFR values before and after transcatheter aortic valve replacement (TAVR), categorized by percent diameter stenosis (%DS). Vessels with baseline %DS < 50% are indicated by solid blue lines, with the dashed blue line representing their mean trend. Vessels with %DS ≥50% are shown by solid red lines, with the dashed red line indicating their mean trend. **(B–D)** Stratified CT-QFR changes following TAVR in the left anterior descending (LAD), left circumflex (LCx), and right coronary (RCA) arteries, respectively, grouped by %DS.

### 3.4 Influence of baseline physiological impairment

Baseline CT-QFR <0.80 was identified in 33 vessels (10.2%). Among these vessels, 23(69.7%) crossed the conventional CT-QFR threshold following TAVR (Figure 3). In contrast, among the 291 vessels with a preoperative CT-QFR ≥ 0.80, only one vessel shifted to a positive CT-QFR status post-TAVR. This single case corresponded to the left circumflex artery, with CT-QFR declining from 0.84 to 0.73. Mixed-effects modeling showed that vessels with baseline CT-QFR <0.80 had substantially lower absolute CT-QFR values than those with baseline CT-QFR ≥0.80 (*β*=−0.203, 95% CI −0.223 to −0.183, *P* < 0.001), as shown in Table 4. However, a significant interaction between TAVR and baseline CT-QFR status was observed (*β*=0.117, *p* for Interaction < 0.001), indicating that vessels with baseline physiological impairment exhibited greater increases in CT-QFR following TAVR (Figures 4, 5).

**Figure 3:**
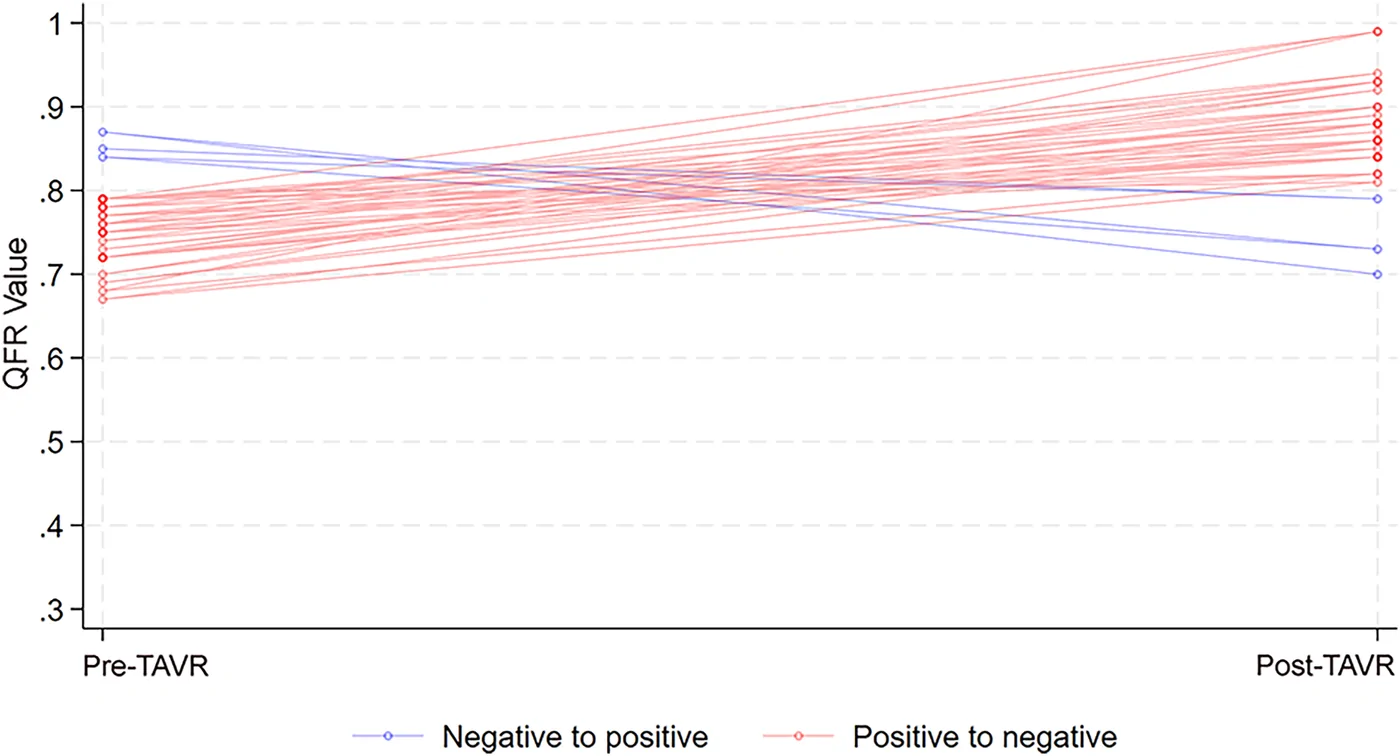
Conversion of CT-QFR values before and after transcatheter aortic valve replacement(TAVR). The blue lines represent Vessels with negative CT-QFR (≥0.8) at baseline converted to positive CT-QFR (<0.8) after TAVR. The red lines represent patients with positive CT-QFR (<0.8) at baseline converted to negative CT-QFR (≥0.8) after TAVR.

**Figure 4:**
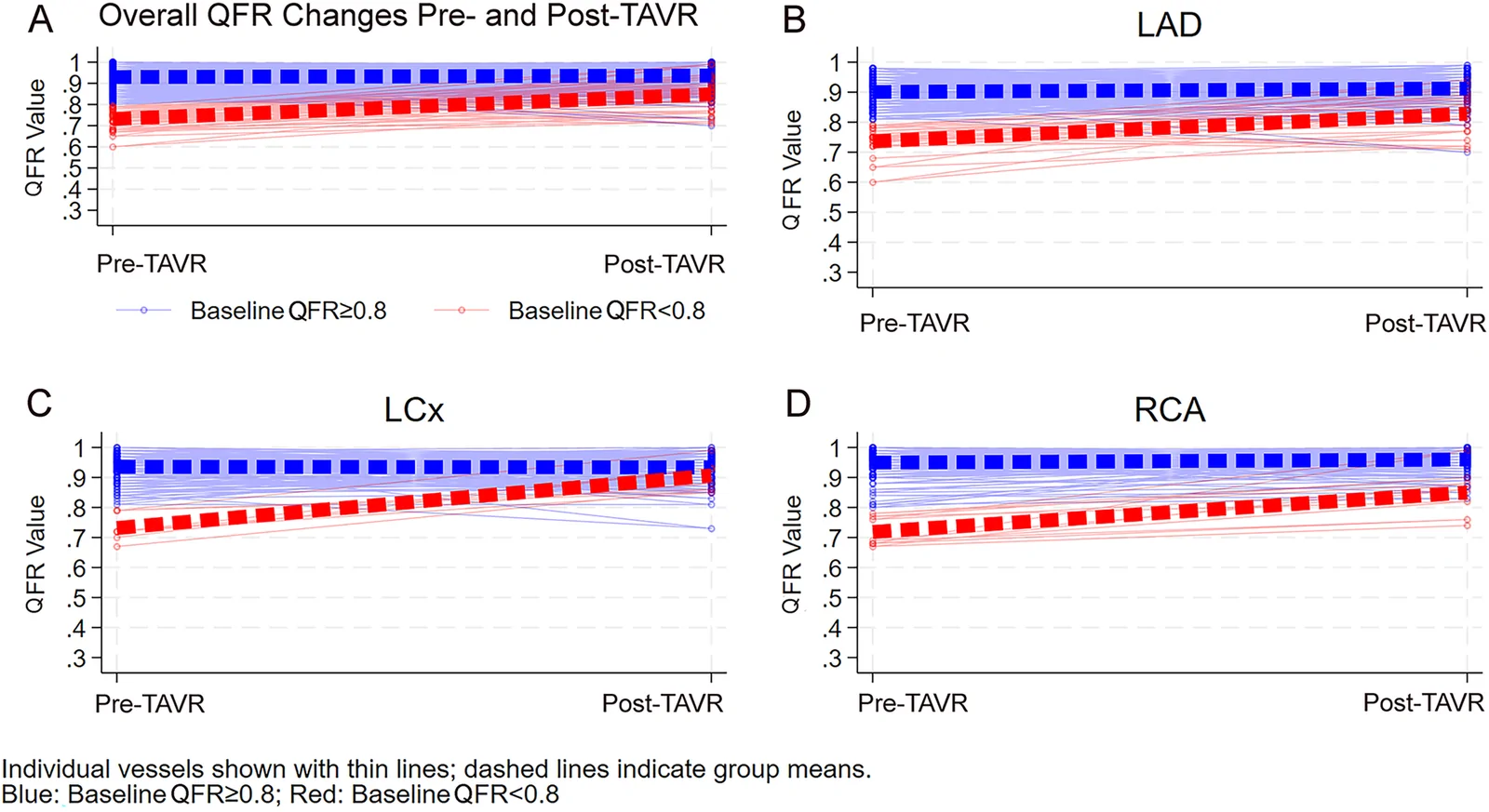
**(A)** overall changes in CT-QFR values before and after transcatheter aortic valve replacement (TAVR) stratified by baseline CT-QFR using a cutoff of 0.8. Vessels with baseline CT-QFR ≥0.8 are indicated by solid blue lines, with the dashed blue line representing their mean trend. Vessels with baseline CT-QFR <0.8 are shown by solid red lines, with the dashed red line indicating their mean trend. **(B–D)** Changes in CT-QFR values before and after TAVR in the left anterior descending artery (LAD), left circumflex artery (LCx), and right coronary artery (RCA), respectively, stratified by baseline CT-QFR.

**Figure 5:**
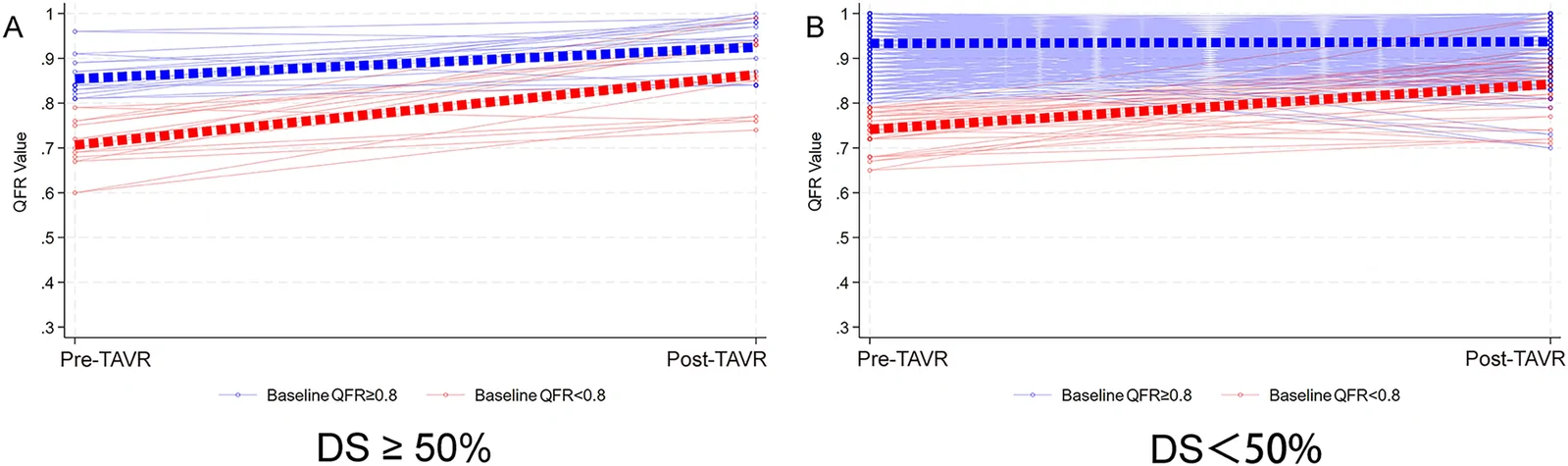
**(A)** the changes of CT-QFR in vessels with %DS ≥50% before and after transcatheter aortic valve replacement (TAVR). Vessels with baseline CT-QFR ≥ 0.8 are indicated by solid blue lines, with the dashed blue line representing their mean trend. Vessels with baseline CT-QFR < 0.8 are shown by solid red lines, with the dashed red line indicating their mean trend. **(B)** The changes of CT-QFR in vessels with %DS <50% before and after TAVR. Vessels with baseline CT-QFR ≥ 0.8 are indicated by solid blue lines, with the dashed blue line representing their mean trend. Vessels with baseline CT-QFR < 0.8 are shown by solid red lines, with the dashed red line indicating their mean trend.

## 4 Discussion

The present study investigated early changes in coronary physiology following TAVR using serial CT-derived quantitative flow ratio assessment and yielded four principal findings. First, TAVR was associated with modest but statistically significant increases in CT-QFR values during the early postprocedural period. Second, these changes were heterogeneous across coronary territories, with significant increases observed in the LAD and RCA, whereas no significant change was identified in the LCX. Third, vessels with greater anatomical stenosis (%DS ≥50%) exhibited larger increases in CT-QFR following valve intervention. Finally, vessels with baseline physiological impairment (CT-QFR <0.80) demonstrated greater improvement in CT-QFR after TAVR.

Collectively, these findings suggest that coronary physiological assessment in severe aortic stenosis may be influenced by relief of valvular obstruction and that CT-QFR measurements obtained before and shortly after TAVR should not necessarily be interpreted as equivalent physiological states.

Severe aortic stenosis profoundly alters coronary hemodynamics through increased left ventricular afterload, elevated myocardial oxygen demand, impaired coronary flow reserve, and microvascular dysfunction (14–16). Although epicardial coronary stenoses may remain anatomically unchanged, the physiological significance of these lesions may vary according to changes in myocardial workload and coronary perfusion conditions. Following TAVR, transvalvular gradients decrease abruptly and ventricular afterload is substantially relieved. In the present study, peak and mean transvalvular pressure gradients decreased markedly after TAVR, confirming successful valve unloading. In contrast, plaque volume, plaque burden, and minimal lumen diameter remained unchanged between examinations, indicating that the observed CT-QFR changes were unlikely to result from alterations in coronary anatomy. Instead, these findings suggest that early CT-QFR changes primarily reflect physiological adaptation following relief of valvular obstruction rather than structural coronary remodeling.

The magnitude of CT-QFR increase observed in the present study was relatively small (overall increase approximately 0.018), and its direct clinical significance remains uncertain. Nevertheless, the consistent direction of change across multiple analyses suggests that coronary physiological measurements in severe aortic stenosis are dynamic and may be influenced by valve intervention.

An interesting finding of the present study was the heterogeneous response among coronary territories. Significant CT-QFR increases were observed in the LAD and RCA, whereas changes in the LCX did not reach statistical significance. Several explanations may account for this observation. The LAD supplies the largest myocardial territory and is therefore particularly sensitive to alterations in myocardial oxygen demand and ventricular wall stress. Relief of pressure overload following TAVR may consequently have a greater influence on physiological flow conditions within the LAD circulation. Similarly, the RCA contributes substantially to myocardial perfusion, particularly in patients with right-dominant coronary anatomy. In contrast, the LCX generally perfuses a smaller myocardial territory and may therefore exhibit less pronounced physiological changes after valve intervention (17). In addition, differences in vessel geometry, coronary dominance, and CT-QFR computational modeling may contribute to inter-vessel variability. Further studies using invasive physiological measurements are required to determine whether these differences represent true biological phenomena or methodological variation.

The present study demonstrated that vessels with greater anatomical stenosis experienced larger CT-QFR increases following TAVR. Similarly, vessels with baseline CT-QFR values below the conventional ischemic threshold of 0.80 showed significantly greater increases than vessels without baseline physiological impairment.

These findings suggest that coronary lesions located closer to the threshold of physiological significance may be particularly sensitive to changes in loading conditions induced by TAVR. Previous invasive studies have demonstrated that indices of coronary physiology may vary substantially before and after relief of severe aortic stenosis, reflecting changes in coronary flow reserve and microvascular resistance rather than changes in epicardial stenosis severity (4, 12, 18).

Importantly, these observations should not be interpreted as evidence that TAVR improves coronary artery disease or reverses myocardial ischemia. Rather, they indicate that the physiological assessment of coronary lesions may be influenced by severe aortic stenosis and subsequent valve intervention. Therefore, coronary functional measurements obtained in the peri-TAVR period should be interpreted with caution, particularly when values are close to clinically relevant decision thresholds.

Several technical considerations should be acknowledged when interpreting CT-QFR values in patients undergoing TAVR.Unlike invasive FFR, CT-QFR estimates hyperemic coronary physiology using computational fluid dynamic models rather than directly measured coronary flow. Severe aortic stenosis is associated with abnormal coronary autoregulation, increased left ventricular filling pressure, myocardial hypertrophy, and microvascular dysfunction (11, 19, 20), all of which may violate assumptions underlying standard CT-QFR computation. Furthermore, rapid changes in transvalvular gradients, systemic blood pressure, myocardial workload, and coronary perfusion pressure immediately following TAVR may alter the hemodynamic environment in which CT-QFR calculations are performed. Metallic prosthetic valve components, residual valvular calcification, and image artifacts may additionally influence coronary segmentation and flow simulation.

Consequently, increases in CT-QFR observed shortly after TAVR should be interpreted as changes in CT-QFR-derived physiological estimates rather than definitive evidence of improved coronary perfusion or myocardial ischemia.

The coexistence of severe aortic stenosis and coronary artery disease remains a frequent and challenging clinical scenario. Determining the optimal timing and necessity of coronary revascularization in patients undergoing TAVR continues to be an area of active investigation.

The present findings suggest that coronary physiological measurements may vary following valve intervention, particularly in lesions with borderline physiological significance or greater anatomical severity. Therefore, coronary functional assessment performed before TAVR may not necessarily predict physiological status after relief of valvular obstruction.

However, the present study should be regarded as hypothesis-generating rather than practice-changing. The current results do not support deferral of coronary revascularization or modification of treatment strategies solely on the basis of expected post-TAVR CT-QFR changes. Prospective studies incorporating invasive physiological measurements and long-term clinical outcomes are required to determine the clinical implications of these observations.

### 4.1 Limitations

Several limitations of this study should be acknowledged. First, this was a single-center retrospective study with a relatively limited sample size, which may restrict generalizability. Second, CT-QFR measurements were obtained during the early postprocedural period, and therefore the long-term evolution of coronary physiology after TAVR remains unknown. Third, invasive physiological measurements such as FFR or iFR were not available for validation of CT-QFR findings. Fourth, CT-QFR is based on computational assumptions that may not fully account for the altered coronary physiology associated with severe aortic stenosis and the immediate post-TAVR state. Finally, the observational design precludes conclusions regarding causality or clinical outcomes.

## 5 Conclusions

TAVR was associated with modest early increases in CT-QFR values, particularly among vessels with baseline physiological impairment or greater anatomical stenosis. These findings suggest that coronary physiological assessment in severe aortic stenosis may be influenced by valve intervention and should therefore be interpreted cautiously during the peri-TAVR period. Further prospective studies are required to determine the clinical significance of these early CT-QFR changes and their potential implications for coronary functional assessment in patients undergoing TAVR.

## Data Availability

The raw data supporting the conclusions of this article will be made available by the authors, without undue reservation.
