# Supplementary material for: Early changes in CT-derived quantitative flow ratio after transcatheter aortic valve replacement: a serial coronary physiological assessment
Source: Front Cardiovasc Med. 2026 Oct 9;13:1902728. doi: 10.3389/fcvm.2026.1902728 (PMC13690769; doi:10.3389/fcvm.2026.1902728)
Supplement: Supplementary file 1 [file Supplementaryfile1.doc]

***Supplementary appendix***

**Fiugure S1.** Patient Selection and Study Flowchart TAVR:Transcatheter aortic valve replacement.

**Figure S2.** ROC curve of diameter stenosis percentage predicting positive CT-QFR (<0.8).

**CTA acquisition**

Scanning was conducted on a third-generation dual-source CT scanner (SOMATOM Force, Siemens Healthineers, Germany). Prior to coronary computed tomography angiography (CCTA), a calcium scoring scan was acquired to evaluate the degree of calcification in each coronary artery. CCTA imaging was carried out using a bolus-tracking technique, with the region of interest positioned in the ascending aorta.

A dual-chamber power injector was used to administer a contrast agent (Iomeprol, Iomeron, 400 mg iodine/mL) intravenously at a flow rate of 4-5 mL/s, followed by a 40 mL saline flush. The volume of contrast media was tailored to each patient’s body weight and the expected scan duration. Tube voltage and current were automatically modulated in real time using automated exposure control based on the patient’s physique.

Image acquisition employed a prospective electrocardiogram (ECG)-gated protocol, with data collected during the 35%-75% phase of the cardiac R-R interval. Reconstruction parameters for the CCTA images included: a detector configuration of 96×0.6 mm, slice thickness of 0.75 mm, reconstruction increment of 0.5 mm, and gantry rotation time of 250 ms. The reference values for tube current and voltage were set at 320 mAs and 100 kVp, respectively.

**CT-QFR calculation**

CT-QFR simulations were conducted using a prototype software system (Cta-Plus; version 2.0, Pulse Medical Imaging Technology, China), which applies a quantitative flow ratio based algorithm. The on-site processing workflow began with automatic segmentation of coronary vessels, with analysis extended to all epicardial vessels reaching a diameter of 2 mm or greater. Following this, the coronary tree was partitioned and organized into a hierarchical structural model. A reference lumen—representing a hypothetical normal vessel segment unaffected by atherosclerotic change—was reconstructed for each vessel.

Patient-specific baseline coronary flow was estimated according to the dimensions of the reconstructed reference lumen and an anisotropic scaling principle. This estimated resting flow was subsequently converted into a simulated hyperemic flow condition. Using this virtual hyperemic flow, CT-QFR values were computed at each point along the coronary tree via a previously validated QFR computational method. Manual corrections of lumen contours were permitted when necessary to ensure simulation accuracy.

For each coronary lesion located in a major epicardial vessel (diameter ≥ 2 mm), the lesion-specific CT-QFR was measured 1-2 cm distal to the stenosis. Vessel-specific CT-QFR was defined as the value at the most distal lesion site. In vessels lacking significant stenosis, the measurement was taken at the farthest distal segment where the vessel diameter remained ≥ 2 mm. The lowest vessel-specific CT-QFR among the major epicardial arteries was used for patient-level assessment, with a value <0.80 indicating hemodynamically significant ischemia.

Two cardiovascular radiologists—with at least 3 years of cardiac imaging experience, respectively—performed all measurements independently, blinded to clinical data and patient outcomes. The final CT-QFR value used in subsequent analysis was the average of the two observers’ measurements.
